# Substrate specificity and function of acetylpolyamine amidohydrolases from *Pseudomonas aeruginosa*

**DOI:** 10.1186/s12858-016-0063-z

**Published:** 2016-03-09

**Authors:** Andreas Krämer, Jan Herzer, Joerg Overhage, Franz-Josef Meyer-Almes

**Affiliations:** Department of Chemical Engineering and Biotechnology, University of Applied Sciences, Haardtring 100, 64295 Darmstadt, Germany; Karlsruhe Institute of Technology (KIT), Institute of Functional Interfaces, 76021 Karlsruhe, Germany

**Keywords:** Acetylpolyamine amidohydrolases, Pseudomonas aeruginosa, Substrate specificity, Acetylpolyamines, Polyamine metabolism

## Abstract

**Background:**

*Pseudomonas aeruginosa*, a Gram-negative, aerobic coccobacillus bacterium is an opportunistic human pathogen and worldwide the fourth most common cause of hospital-acquired infections which are often high mortality such as ventilator-associated pneumoniae. The polyamine metabolism of *P. aeruginosa* and particularly the deacetylation of acetylpolyamines has been little studied up to now. Results with other bacterial pathogens e.g., *Y. pestis* suggest that polyamines may be involved in the formation of biofilms or confer resistance against certain antibiotics.

**Results:**

To elucidate the role of acetylpolyamines and their enzymatic deacetylation in more detail, all three putative acetylpolyamine amidohydrolases (APAHs) from *P. aeruginosa* have been expressed in enzymatic active form. The APAHs PA0321 and PA1409 are shown to be true polyamine deacetylases, whereas PA3774 is not able to deacetylate acetylated polyamines. Every APAH can hydrolyze trifluoroacetylated lysine-derivatives, but only PA1409 and much more efficiently PA3774 can also process the plain acetylated lysine substrate. *P. aeruginosa* is able to utilize acetylcadaverine and acetylputrescine as a carbon source under glucose starvation. If either the PA0321 or the PA1409 but not the PA3774 gene is disrupted, the growth of *P. aeruginosa* is reduced and delayed. In addition, we were able to show that the APAH inhibitors SAHA and SATFMK induce biofilm formation in both PA14 and PAO1 wildtype strains.

**Conclusions:**

*P. aeruginosa* has two functional APAHs, PA0321 and PA1409 which enable the utilization of acetylpolyamines for the metabolism of *P. aeruginosa.* In contrast, the physiological role of the predicted APAH, PA3774, remains to be elucidated. Its ability to deacetylate synthetic acetylated lysine substrates points to a protein deacetylation functionality with yet unknown substrates.

**Electronic supplementary material:**

The online version of this article (doi:10.1186/s12858-016-0063-z) contains supplementary material, which is available to authorized users.

## Background

*Pseudomonas aeruginosa,* a versatile Gram-negative bacterium, is an opportunistic human pathogen that is worldwide the fourth most common cause of hospital-acquired infections of the gastrointestinal, urinary or respiratory tracts. These infections often result in fatal courses of disease. The emergence of *P. aeruginosa* as one of the most important nosocomial pathogens correlates with increasing resistance to antibiotics and disinfectants as well as the formation of highly resistant biofilms. *P. aeruginosa* has one of the most versatile metabolic arsenals of any described bacterium including its understudied polyamine metabolism [1]. Polyamines are positively charged small organic molecules that are widely distributed and occur at high concentrations in the millimolar range in nearly all prokaryotic and eukaryotic cells but also extracellularly e.g., in human serum or plasma. Polyamines are known to play pivotal roles in many cellular processes including stabilization of DNA, regulation of DNA-protein interaction, posttranslational modification, cell cycle regulation, differentiation and apoptosis [2]. In prokaryotes polyamines are implicated in oxidative stress responses [3], biofilm formation [4–6] and antibiotic resistance [7, 8]. It is therefore not surprising that polyamines, their biosynthesis and transport systems are regarded as possible virulence factors of important human bacterial pathogens [9–12]. Particularly for *P. aeruginosa*, polyamines were shown to induce a complex yet not completely understood resistance mechanism against cationic peptide, aminoglycoside and quinolone antibiotics which was linked to the polyamine metabolism [7]. The role of polyamines in biofilm formation of *P. aeruginosa* is still unknown. But the metabolism of agmatine, a precursor of putrescine, was shown to be linked to the development of a biofilm which let the authors hypothesize that preferential induction of the agu2ABCA’ operon containing two genes for agmatine deiminases by agmatine in the stationary phase and during biofilm growth may have evolved to provide polyamines for biofilm development [6]. Although polyamines are required for growth of *P. aeruginosa*, these compounds could be toxic in high excess. Therefore, polyamine homeostasis must be maintained through a fine regulated network of polyamine biosynthesis, catabolismus, excretion and uptake. Excess polyamine in many types of bacteria, including *E. coli, Bacillus subtilis* and *S. aureus,* is acetylated, thereby converted into a physiologically inert form and subsequently excreted to maintain the polyamine level [13]. In contrast, *P. aeruginosa* possesses no homolog of the respective acetyltransferase in *E. coli* as revealed by sequence similarity search. Chou et al. hypothesize that polyamine homeostasis in *P. aeruginosa* is kept mainly through two catabolic pathways [14]. The polyamine putrescine is converted into 4-aminobutyrate (GABA) either via the conserved transamination and dehydrogenation route or the γ-glutamylation route [15]. Yao et al. postulate six γ-glutamylpolyamine synthetases to initiate polyamine catabolism and suggest them as a molecular target for new antibiotic strategies exploiting the alleviation of polyamine toxicity when in excess [12]. Only few studies reported on polyamine transporters. One of them was identified by Lu et al. and proposed to be an ABC transporter system for spermidine uptake [16]. In addition, this polyamine transport system was linked to the type III secretion system, which is a major virulence factor in bacteria [17]. The molecular recognition of polyamines by the transporter system was elucidated by Wu et al. providing a rational approach to blocking type III secretion through targeting of the polyamine uptake system [18]. A similarity search for homologous sequences of histone deacetylase enzymes revealed three genes for putative acetylpolyamine amidohydrolases (APAHs) in the genome of *P. aeruginosa* PA01 [19]. Like other bacterial APAHs, e.g., from *Mycoplana ramosa*, the corresponding protein sequences from *P. aeruginosa* belong to the histone deacetylase family, and the amino acids lining the active site and chelating the catalytic zinc ion are highly conserved. As pointed out above, no similar sequences to a polyamine acetyltransferase could be found in the *P. aeruginosa* genome. Therefore, the specific role of the predicted APAHs appears to be unclear. In the following, the putative APAH enzymes are named after their gene designation, i.e., PA0321, PA1409 and PA3774. The function of these enzymes has been only sparsely investigated before. PA3774 was shown to be closely related to HDAH and able to hydrolyze an artificial acetylated lysine substrate [20]. On the base of transcriptome data and the chemical similarity between N-carbamoyl- and N-acetylputrescine, PA1409 and PA0321 have been proposed to be involved in the conversion of agmatine into putrescine [14]. This statement was underlined by the induction of the genes of PA0321 and PA1409 by exogenous acetylputrescine and agmatine which was suggested to be mediated by N-carbamoyl-putrescine. However, only the deacetylation of acetylputrescine by PA1409 was proven with a purified enzyme. Given this fact, it remained an open question whether acetylputrescine or other acetylated polyamines may be the natural substrates of the three putative APAHs from *P. aeruginosa* even though no obvious opponent polyamine acetyltransferases are identified yet. Herein, we perform a combined biochemical and microbiological study to dissect the substrate specificity of the three putative APAHs in *P. aeruginosa* and suggest probable roles of these enzymes in the living organism.

## Results and discussion

### The putative APAHs of *P. aeruginosa* are members of the histone deacetylase family

**Fig. 1:**
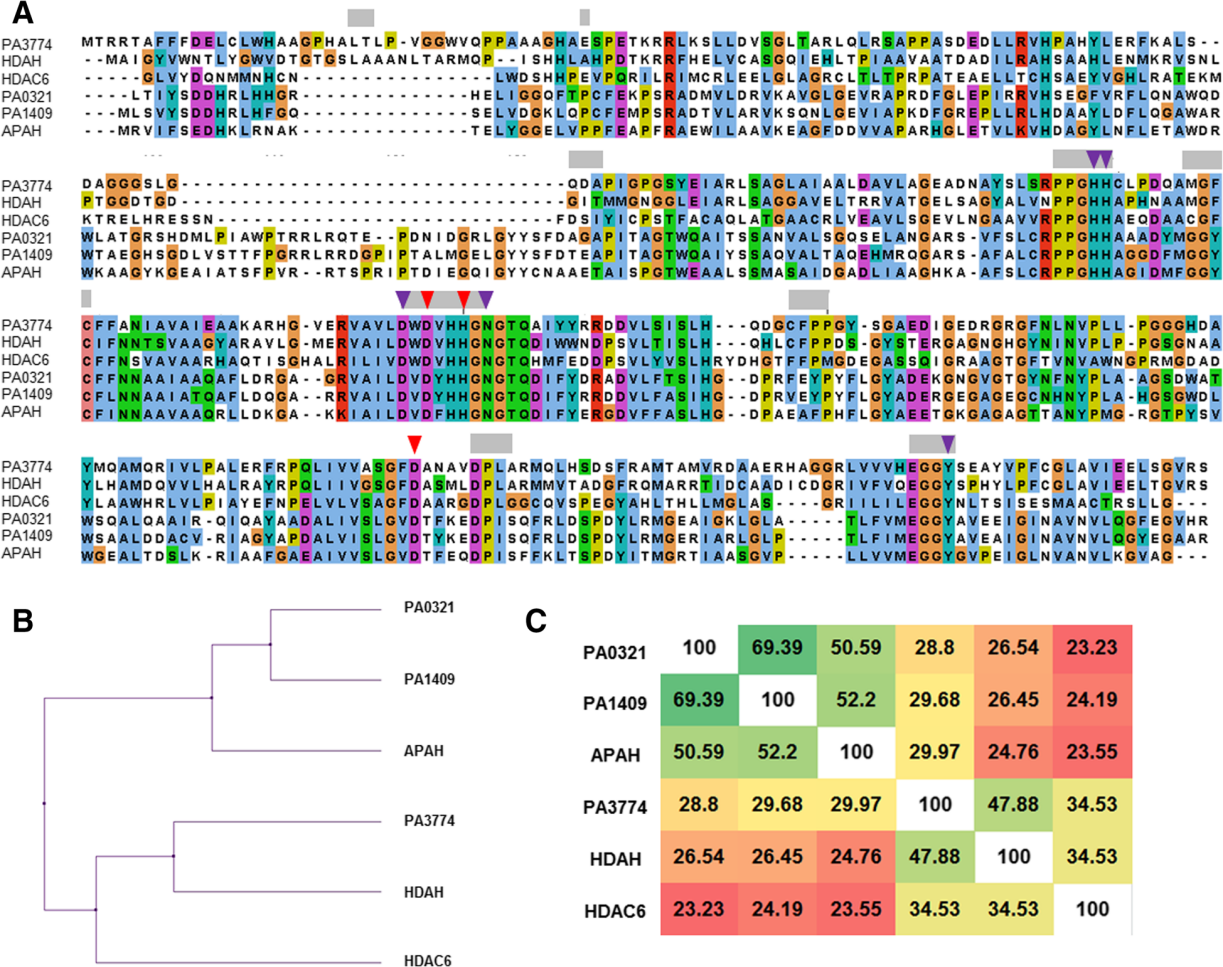
**a** Multiple Sequence Alignment of PA0321, PA1409 and PA3774 from *P. aeruginosa*, acetylpolyamine amidohydrolase APAH from *M. ramosa*, histone deacetylase like amidohydrolase HDAH from *Bordetella sp.* and human HDAC6 (*second deacetylase domain*). The red triangles mark the amino acids that complex the catalytic Zn^2+^-ion and the magenta triangles the amino acids involved in the catalytic mechanism. The bars in light gray denote amino acids lining the binding pocket. **b** Average distance tree calculated from MSA in A) using the BLOSUM62 similarity matrix. **c** Percent identity matrix calculated using ClustalW2. Similarities between sequences are colored from non-identical high (*green*) to low (*red*)

The role of polyamine metabolismus in the formation of pathogenic biofilms [4–6, 21] inspired our search for novel enzymes, particularly deacetylases which potentially take part in the polyamine metabolism of *P. aeruginosa*. Exploiting the *Pseudomonas* Genome Database [22] three putative APAHs were found: PA0321, PA1409 and PA3774 which show remarkable sequence similarity to enzymes belonging to the histone deacetylase family. The amino acid sequences of these enzymes were subjected to a Multiple Sequence Alignment including HDAH from *Bordetella sp.*, APAH from *M. ramosa* and human HDAC6. The bacterial enzymes HDAH, APAH from *M. ramosa* and human HDAC6 were selected due to their high similarity to PA3774 and PA1409 or PA0321, respectively. In addition, the 3D-structures of HDAH [23] and APAH from *M. ramosa* [24] are available and allowed a mapping of the amino acids of the enzymes from *P. aeruginosa* to their function and position with respect to the active site. The essential amino acids discussed in the following are numbered according to the X-ray structure of HDAH (PDB 1ZZ1). D180, D268 and H182 are responsible for chelating the catalytic Zn^2+^-ion and are identical for all aligned proteins (Fig. 1a). Likewise, the amino acids involved in the catalytic mechanism (Y312, H142, H143, D178, N185), are the same in all sequences [25, 26]. In addition, the channel to the catalytic center is lined with highly homologous amino acids (Fig. 1a). Based on their sequence, PA0321 and PA1409 form a cluster with the verified functional acetylpolyamine amidohydrolase APAH from *M. ramosa*, whereas PA3774, HDAH and HDAC6 are grouped in a different cluster (Fig. 1b). HDAC6 was chosen because it is the closest human homolog to PA3774. In fact, PA0321 and PA1409 are the enzymes with highest percent identity (69.39 %) and still high identity of 50.59 and 52.2 % to the APAH from *M. ramosa* (Fig. 1c). The closest homolog of PA3774 is HDAH whose natural substrate is still unknown. HDAH has been shown to deacetylate proteins, e.g., histones, although it is a bacterial enzyme. Both, PA3774 and HDAH, show a moderate percent identity of 34.53 % to the second domain of human HDAC6, a proven deacetylase of several cytosolic proteins [27–29]. The sequence based distinct clustering of the three deacetylases from *P. aeruginosa* with APAH from *M. ramosa* or HDAH and HDAC6 suggested a true acetylpolyamine amidohydrolase functionality of PA0321 and PA1409. In contrast, the role of PA3774 appeared to be different. The similarity to protein deacetylases gave rise to the assumption that yet unknown proteins rather than polyamines would be substrates of PA3774.

### In-vitro substrate-specificity of the putative APAHs from *P. aeruginosa*

**Fig. 2:**
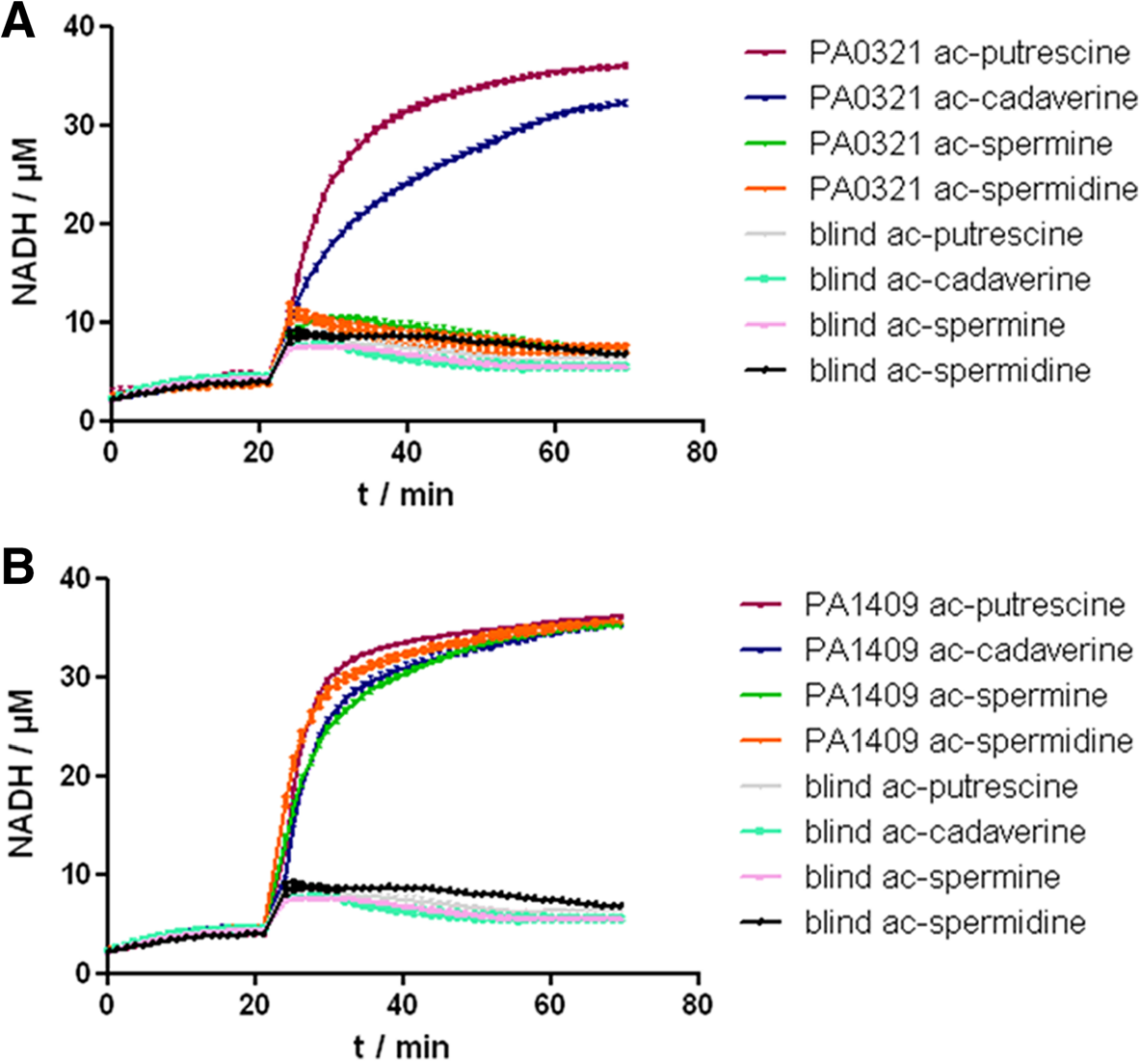
Acetate release assay: The indicated acetylpolyamines were added to **A**) PA0321 and **B**) PA1409 after the equilibrium of the assay mix was formed (ca. 22 min) the activities were calculated from the linear increase in NADH concentration after adding the acetylated polyamines. Protein concentrations were 200 nM for PA0321 and 100nM for PA1409

The putative APAHs from *P. aeruginosa*, PA0321, PA1409 and PA3774, have already been expressed in *E. coli* previously [14, 20, 30], however, a detailed characterization with respect to function and substrate specificity is still missing. PA1409 was shown to deacetylate acetylputrescine in a transcriptome analysis of agmatine and putrescine catabolism [14], and the functionality of PA3774 as closest homolog of HDAH from *Bordetella sp.* was only investigated with fluorogenic lysine substrates that were either trifluoroacetylated, acetylated or propylated at the ε-amino group of lysine with decreasing maximal enzyme velocities [20]. In contrast, PA0321 was functionally characterized for the first time in this study. The substrate specificity of all above mentioned putative APAHs from *P. aeruginosa* was analyzed using a panel of four acetylated polyamines and two lysine-based fluorogenic substrates. Two orthogonal assay systems were applied to measure the deacetylation of acetylpolyamines. The diamine oxidase assay was only applicable to the shorter acetylcadaverine and acetylputrecine but not to N^1^-acetylspermine or N^1^-acetylspermidine, because the latter substrates already generate an assay signal without being deacetylated by one of the APAHs. For comparison and to investigate the hydrolysis of N^1^-acetylspermine and N^1^-acetylspermidine, which could otherwise not be examined, a more general optimized acetate release assay was exploited [31]. The diamine oxidase assay proved that both, acetylcadaverine and acetylputrescein are deacetylated by PA0321 and PA1409 but not by PA3774 (Table 1). Both, PA0321 and PA1409, exhibit K_M_-values between 0.2 and 0.5 mM which is about one order of magnitude lower than the typical intracellular bacterial polyamine concentrations [4]. PA1409 appears to be the more active enzyme and reaches about the three-fold maximum velocity of PA0321 for the hydrolysis of the small acetylpolyamines in the quantitative diamine oxidase assay. The acetate release assay confirmed that PA0321 and PA1409 could hydrolyze acetylcadaverine and acetylputreseine, whereas PA3774 could not (Table 1, Fig. 2). In addition, it could be shown that N^1^-acetylspermine and N^1^-acetylspermidine were both substrates of PA1409, whereas PA0321 showed only very low catalytical activity to deacetylate N^1^-acetylspermine. Taken together, PA0321 and PA1409 are efficient APAHs with different selectivity for acetylated polyamines with PA1409 being the more active and less selective enzyme. Moreover, the high sequence homology of both enzymes from *P. aeruginosa* and the APAH from *M. ramosa* (Fig. 1) is reflected by a comparable broad acetylpolyamine substrate tolerance [32]

**Table 1:** Enzyme activity parameters using different substrates

Enzyme		Ac-Cad	Ac-Put	N^1^-Ac-Spermine	N^1^-Ac-Spermidine	Boc-K-(Ac)-AMC	Boc-K-(Tfa)-AMC
PA0321	K_M_/μM	284 ± 39^c^	411 ± 34^c^	^e^	^e^	22 ± 10	11 ± 1
	V_max_/(nmole mg^−1^ s^−1^)	7.6 ± 0.3^c^	14.2 ± 0.4^c^	^e^	^e^	0.017 ± 0.006	21 ± 2
	V/(nmole mg^−1^ s^−1^)	2.9 ± 0.1^d^	4.9 ± 0.2^d^	≤0.001^d^	≤0.001^d^	-	-
PA1409	K_M_/μM	465 ± 53^c^	503 ± 60^c^	^e^	^e^	223 ± 12	84 ± 15
	V_max_/(nmole mg^−1^ s^−1^)	24.5 ± 0.9^c^	39.3 ± 1.5^c^	^e^	^e^	1.3 ± 0.1	5.2 ± 0.6
	V/(nmole mg^−1^ s^−1^)	11.1 ± 0.3^d^	10.2 ± 0.1^d^	7.4 ± 0.1^d^	7.2 ± 0.2^d^	-	-
PA3774	K_M_/μM	NA^c^	NA^c^	^e^	^e^	95 ± 7	21 ± 2
	V_max_/(nmole mg^−1^ s^−1^)	≤0.01^c^	≤0.01^c^	^e^	^e^	3.4 ± 0.1	8.6 ± 0.3
	V/(nmol mg^−1^ s^−1^)	≤0.001^d^	≤0.001^d^	≤0.001^d^	≤0.001^d^	-	-
HDAH	K_M_/μM	-	-	-	-	26 ± 4	7.2 ± 1.1
	V_max_/(nmole mg^−1^ s^−1^)	0.18^a^	0.13^a^	-	0.06^a^	0.43 ± 0.02	0.23 ± 0.01
APAH *M. ramosa*	K_M_/μM	50^b^	220^b^	290^b^	130^b^	-	-
	V_max_/(nmole mg^−1^ s^−1^)	450^b^	450^b^	223^b^	275^b^	-	-

*NA* cannot be determined due to a very low signal, ^a^ values were taken from [46], ^b^ values were taken from [32], assay was performed at 30 °C, ^c^ this study: diamine oxidase assay performed at 21 °C, ^d^: this study: acetate release assay at 200 μM substrate concentration, where V_max_ is not reached, ^e^: not compatible with diamine oxidase assay. Mean values and standard errors are based on at least three replicates

It was also instructive to examine the enzyme activity of the deacetylases from *P. aeruginosa* using fluorogenic lysine substrates usually used to assay human histone deacetylases. The chemically activated Boc-K-(TFAc)-AMC substrate was processed by all enzymes with different V_max_ values. More distinct differences occurred using the plain Boc-K-(Ac)-AMC substrate: PA0321 showed the lowest and PA3774 the highest activity under standard assay conditions (Additional file 1: Figure S1). Therefore, sequence homology and in vitro substrate specificity point in the same direction and suggest that PA0321 and PA1409 are predominantly true acetylpolyamine amidohydrolases whereas PA3774 is clearly not. The pronounced capability of PA3774 to process both lysine-based substrates lets us hypothesize that its principal task is the deacetylation of yet unknown protein substrates.

### Growth analyses and biofilm formation

**Fig. 3:**
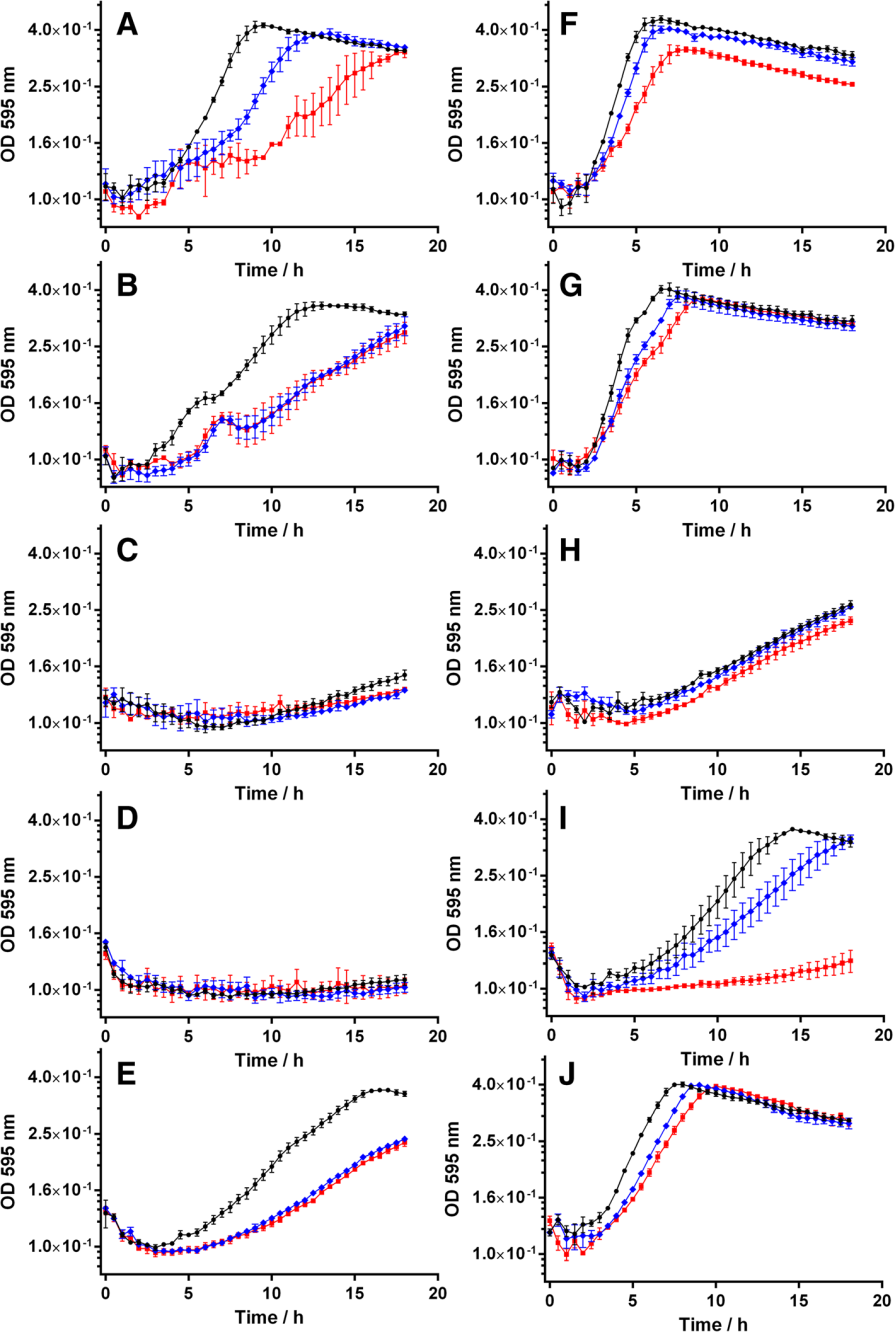
Growth curves of *P. aeruginosa* wildtypes (PA01: **a**/**f**, PA14: **b**/**g**) and mutants with defective PA0321 (**c**/**h**), PA1409 (**d**/**i**) or PA3774 (**e**/**j**) gene, respectively. All growth curves were performed in the absence of glucose and in the presence of 10 mM acetylcadaverine (*first column*) or 10 mM acetylputrescine (*second column*). All test conditions were applied in the absence (*black circles*), in the presence of 50 μM SAHA (*blue diamond*) or in the presence of 50 μM SATFMK (*red squares*). The data points represent three biological replicates with indicated standard error

To unravel the physiological function of the three deacetylases PA0321, PA1409 and PA3774 in vivo, the two wildtype strains, PA01 and PA14 as well as the three PA14 transposon mutant strains ΩPA0321, ΩPA1409, and ΩPA3774, each with a disrupted gene for one single deacetylase, were investigated by analyzing their growth kinetics in culture media supplemented with acetylcadaverine or acetylputrescine, respectively. In the presence of glucose together with either acetylcadaverine or acetylputrescine, the inhibitors SAHA and SATFMK have no influence on the growth curves of PA01 and PA14 wildtype strains (Additional file 1: Figure S2) indicating, that none of the deacetylases possesses a vital function in the presence of an ample alternative carbon source. However, using acetylcadaverine or acetylputrescin polyamines as the only carbon source, a significant growth reduction was detected for PA01 and to a lesser extent for PA14 cultures in the presence of SAHA and SATFMK acetylase inhibitors, respectively, with a stronger effect for SATFMK (Fig. 3 a, b, f, and g). In the presence of acetylcadaverine, the ΩPA1409 mutant was unable to use this polyamine as a substrate within the analyzed period of time (Fig. 3 d) or had an extreme growth retardation. The ΩPA0321mutant showed also a markedly delayed growth with a slight hint of increase after 15 h (Fig. 3 c). In contrast, the ΩPA3774 mutant regained the capability to utilize acetylcadaverine similar to the PA14 wildtype (Fig. 3 e). This suggests, that nor PA014 neither PA0321 on its own can efficiently metabolize acetylcadaverine, only if both enzymes are intact (but PA3774 not), acetylcadaverine can be utilized as a substrate. The effects become clearer with another polyamine substrate. Using acetylputrescine as sole carbon source for growth of ΩPA0321 and ΩPA1409, we detected an elongated lag-phase of approximately 6 h before both mutant strains grew (Fig. 3 h, and i). For the ΩPA0321 mutant no significant influence of SAHA and SATFMK could be detected. In contrast, growth of the ΩPA1409 mutant in the presence of acetylputrescine could be completely inhibited by SATFMK and significantly by SAHA. Growth of the ΩPA3774 mutant in the presence of both acetylcadaverine and acetylputrescine was comparable to growth of the PA14 wildtype (Fig. 3 b, g, e and j). This finding is in line with the biochemical results showing that only PA0321 and PA1409 but not PA3774 can convert acetylated cadaverine and putrescine into the corresponding unmodified polyamines which can be further metabolized by the bacterium. The extent to which the growth of the mutants is inhibited also correlates with the biochemical in vitro potency of the applied inhibitors. The growth of the ΩPA1409 mutant containing intact PA0321 in the presence of acetylputrescine can be completely inhibited by SATFMK which is more than 40-times potent against PA0321 compared with PA1409 [30] (Fig. 3 i). On the other hand, growth of the PA0321 mutant in medium supplemented with acetylputrescine could only be inhibited slightly, probably due to the lower potency of SATFMK against PA1409 [30]. This may also explain to some extent, why growth of the PA01 and PA14 wildtypes and mutant strains of *P. aeruginosa* with functional PA1409 cannot be completely inhibited by SAHA or SATFMK. Altogether, the growth curve experiments agree well with the biochemical in vitro results and provide evidence that PA0321 and PA1409 but not PA3774 play an important role to the acetylated polyamines metabolism in *P. aeruginosa*.

**Fig. 4:**
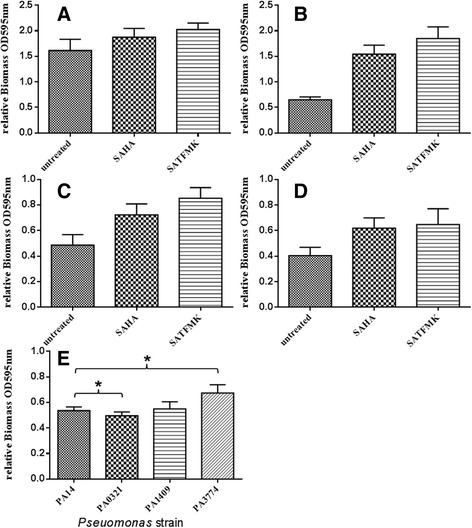
Relative biomass OD_595nm_ of *Pseudomonas* PA01 and PA14 strains in BM2 media with 10mM acetylcadaverin or acetylputrescin polyamines as sole carbon source, challenged with 50 μM deacetylase inhibitors SAHA or SATFMK. **a** PA14 strain with acetyl-cadaverine as carbon source (**b**) PA14 strain with acetylputrescine as carbon source (**c**) PA01 strain with acetylcadaverine as carbon source (**d**) PA01 strain with acetylputrescine as carbon source. **e** Biofilm induction of PA14 mutant strains in the presence of 0.5 % (w/v) casamino acids and in the absence of HDAC inhibitors. Biomass was quantified after 24h incubation in a static biofilm assay (*n* = 18)

In addition to planktonic growth, microorganisms usually grow as surface-attached biofilms, which provide the bacteria protection against antibiotics or the host immune system [33] and play therefore a crucial role in many chronic infections [34]. This encouraged us to analyze the impact of the deacetylase inhibitors SAHA and SATFMK and the deacetylase gene knock-outs on biofilms of *P. aeruginosa*. Using acetylcadaverine or acetylputrescine as sole carbon source in combination with the deacetylase inhibitors SAHA or SATFMK, respectively, we detected a stimulating effect of both inhibitors on biofilm formation of the PA01 and PA14 wildtype, with the strongest effect in the presence of SATFMK (Fig. 4a–d). In order to assess wether the disruption of a single deacetylase also impacts on biofilms of *P. aeruginosa*, we analyzed biofilm formation of the mutant strains ΩPA0321, ΩPA1409, and ΩPA3774 in the presence of 0.5 % (w/v) casamino acids which induces biofilm formation in *P. aeruginosa*. In comparison to the wildtype PA14, the mutant strains ΩPA0321 and ΩPA1409 exhibited only marginal changes in biofilm biomass. In contrast, the ΩPA3774 mutant showed a more significant attenuation in biofilm formation with a 25 % increase in biofilm biomass after 24 h of incubation compared to wildtype cells (Fig. 4e). Due to the fact that biofilm formation is a rather complex processes influenced by a large number of different proteins and factors [35], the exact mechanism of how knock-out of PA3774 as well as the addition of SAHA and SATFMK impacts on biofilm formation in *P. aeruginosa* is unknown and will be investigated in more detail in the future. However, since a large number of studies have shown, that environmental stressors e.g., ethanol [33] or the antibiotics tetracycline, clarithromycin, ciprofloxacin, imipenem or gentamicin [34, 36–38] can induce biofilm formation in *P. aeruginosa*, the stimulating effects of SAHA and SATFMK on biofilm formation could reflect such a response to cellular stress for *P. aeruginosa.*

## Conclusions

Biochemical and growth experiments demonstrate that PA0321 and PA1409 are functional acetylpolyamine amidohydrolases and are able to make acetylated polyamines accessible for the metabolism of *P. aeruginosa* under lack of other carbon sources. In contrast, the postulated APAH PA3774 does not hydrolyze acetylpolyamines and shows no detectable influence on the growth of *P. aeruginosa*. The function of PA3774 remains elusive, but, based on sequence homology, the enzyme is a member of the histone deacetylase superfamily and effectively hydrolyzes acetylated and trifluoroacetylated lysine substrates suggesting a possible role as lysine deacetylase of hitherto unknown protein substrates. SAHA and SATFMK inhibit all three deacetylases from *P. aeruginosa*, and show a stimulating impact on biofilm formation by an unknown mechanism.

## Methods

All basic chemicals were purchased at the highest available purity level from Sigma-Aldrich, Merck, ROTH, Acros Organics or Applichem. The substrates, enzymes and inhibitors used in the assays were obtained from the following suppliers: N-(5-aminopentyl) acetamide (acetylcadaverine) and N-(4-aminobutyl) acetamide (acetylputrescine) from TCI, N^1^-Acetylspermine from FLUKA, N^1^-Acetylspermidine and SAHA from Cayman Chemical Company,

Boc-K-(Tfa)-AMC and Boc-K-(Ac)-AMC from Bachem, citrate synthase, diamine oxidase and horseradish peroxidase type 1 from Sigma-Aldrich, trypsin from Applichem and the malatdehydrogenase from Calbiochem. SATFMK was synthesized according to [39]. PA0321, PA1409 and PA3774 were recombinantly produced as described before [30]. The acetyl-CoA synthetase was recombinantly expressed and purified according to Fierke et al. [31] using a plasmid that was kindly provided by the cited working group.

### Boc-K-(Ac/Tfa)-AMC assay

Lysine deacetylase activity was measured using the well described Boc-K-(Ac/Tfa)-AMC assay [40]. Briefly, the cleavage of trifluoroacetate or acetate by the enzyme allows trypsin to cleave the fluorophore from the substrate resulting in a dramatic increase in fluorescence (Ex.: 340 nm, Em.: 460 nM). The tests were run at 30°C with 10–100 nM enzyme and 0.5 mg/mL trypsin dissolved in assay buffer (20 mM Tris–HCl pH 8, 50 mM NaCl, 0.001 % Pluronic). Because trypsin doesn’t digest the deacetylases the test could performed in a continuous manner. For Michelis-Menten kinetics the Boc-K-(Tfa)-AMC was varied from 0 to 80 μM (maximum solubility) and the Boc-K-(Ac)-AMC from 0 to 250 μM. The experiment was carried out in a PheraStar Fluorescence Spectrometer (BMG Labtech). The data were analyzed with the program Graph Pad Prism.

### Acetate assay

Briefly, it is an enzyme-coupled assay which can measure the release of acetate directly by coupling to the formation of NADH. This has the advantage of working with potential native substrates [31]. Measurements were carried out at 30°C with an enzyme concentration of 100 nM in assay buffer and final concentrations of 50 mM HEPES pH 8, 127 mM NaCl, 2,7 mM KCl, 6 mM MgCl_2_, 1,25 mM L-malic acid, 200 μM ATP, 5 μM NAD^+^, 15 μM CoA, 0,02 U/μL malat dehydrogenase, 0,04 U/μL citrate-synthase and 25 μM acetyl-CoA-synthetase. This master mix was incubated until equilibrium (ca. 30 min) and then the reaction was initiated by adding either 200 μM acetylputrescine, acetylcadavarine, N^1^-acetylspermine or N^1^-acetylspermidine to the mixture. The time-dependent increase in NADH fluorescence (Ex = 340 nm, Em = 460 nm) corresponds to the deacetylase activity.

### Diamine oxidase assay

Alternatively, acetylpolyamine deacetylase activity was measured using a modified colorimetric assay that quantifies putrescine or cadaverine generation by converting it to γ-aminobutyraldehyde and hydrogen peroxide using diamine oxidase and then utilizing the hydrogen peroxide product in reaction with vanillic acid and 4-aminoantipyrene to generate quinoneimine (absorbance at λ = 498 nm) [24, 41]. For Michelis-Menten kinetics the concentration of acetylcadaverine and acetylputrescine was varied from 0 to 25 mM in assay buffer containing 100mM HEPES (pH7.4), 10 mM KCl, 4 mM CaCl_2_, 2.8 mM MgCl_2_ and 280 mM NaCl. The reaction was started by adding the master mix containing 0.5 mM 4-aminoantipyrene, 1 mM vanillic acid, 4 U/mL horseradish peroxidase, 1 U/mL diamine oxidase and 100 nM of the desired protein dissolved in assay buffer. The assay was run in a continuous manner using a TECAN Genious Spectra FLUOR Plus reader. The activity was calculated from the slope of the rising absorbance at at 498 nm minus any background at the same concentration without protein. The K_M_ and V_max_ values were calculated using the the Program GraphPad Prism.

### Growth curves and biofilm quantification

The *Pseudomonas aeruginosa* strains PA01 [19], PA14 and the respective PA14 transposon insertion mutants ΩPA0321 (ID: 47664), ΩPA1409 (ID: 53313) and ΩPA3774 (ID: 39836) [42] were used to analyze the influence of acetylcadaverine and acetylputrescine polyamines on growth and biofilm phenotypes. Bacteria were routinely grown in BM2 media (62 mM potassium phosphate buffer (pH 7), 7 mM (NH_4_)_2_SO_4_, 2 mM MgSO_4_, 10 μM FeSO_4_, Glucose 0.4% (w/v) [43]. When mentioned in the text, Glucose was replaced by 10 mM polyamines (acetylcadaverine or acetylputrescine) as the only carbon source. The deacetylase inhibitors SAHA or SATFMK were used at a final concentration of 50 μM. Bacteria were cultivated in 96-well polystyrene microtiter plates (Nunc, Thermo Fisher Scientific) with 100 μl culture volume per well in a microtiter plate reader (Tecan Group Ltd.). Static biofilms were grown as described previously [44]. The biofilm biomass was stained after 24 h of static incubation at 37°C with crystal violet [45] and the optical density was subsequently measured at 595 nm absorbance. For growth curves the optical density (OD_595nm_) was measured for 18 h at 37°C under shaking conditions in a microtiter plate reader (Tecan Group Ltd.). Growth curve analyses were performed at least in triplicate and biofilm analyses at least in triplicate with multiple wells per condition.

## Additional file

Additional file 1: Figure S1. Michaelis-Menten kinetics. **Figure S2.** Impact of SAHA and SATFMK on the growth of *P. aeruginosa* strain PA01 and PA14 in the presence of glucose. (DOCX 371 kb)
