# Supplementary material for: Substrate specificity and function of acetylpolyamine amidohydrolases from Pseudomonas aeruginosa
Source: BMC Biochem. 2016 Mar 9;17:4. doi: 10.1186/s12858-016-0063-z (PMC4784309; doi:10.1186/s12858-016-0063-z)
Supplement: Additional file 1: Figure S1. — Michaelis-Menten kinetics. Figure S2. Impact of SAHA and SATFMK on the growth of P. aeruginosa strain PA01 and PA14 in the presence of glucose. (DOCX 371 kb) [file 12858_2016_63_MOESM1_ESM.docx]

Substrate Specificity and Function of Members of the Histone Deacetylase Family from *Pseudomonas aeruginosa*

Andreas Krämer^1^, Jan Harzer^2^, Joerg Overhage^2^ and Franz-Josef Meyer-Almes^1^*

**Fig. S1: Michaelis-Menten kinetics**

# Fig. S2: Impact of SAHA and SATFMK on the growth of *P. aeruginosa* strain PA01 and PA14 in the presence of glucose


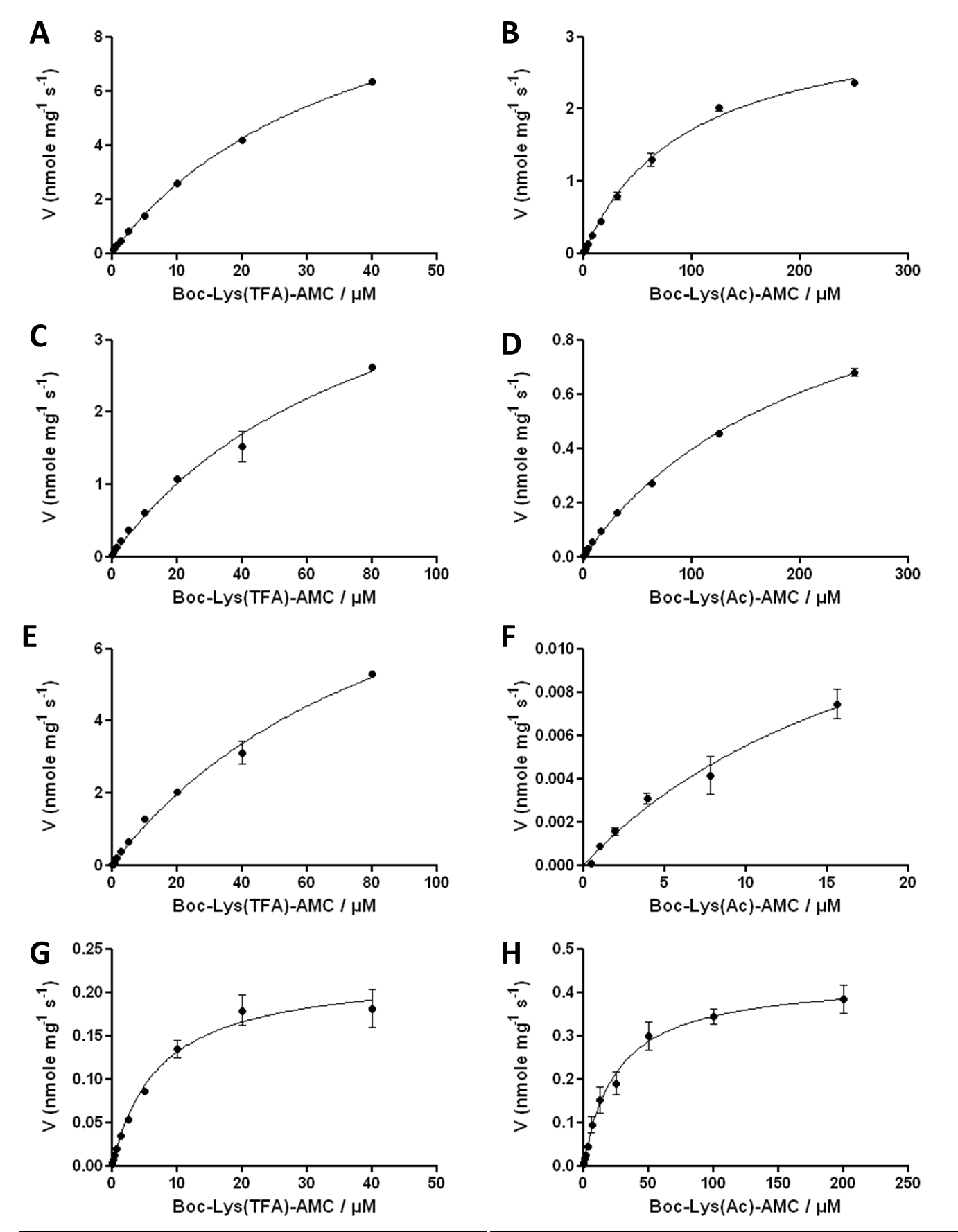


Fig. S1: Michaelis-Menten kinetics of lysine-deacetylation of PA3774 (A and B), PA1409 (C and D), PA0321( E and F) and HDAH (G and H): The deacetylation kinetics of the denoted fluorogenic acetylated (Ac) or trifluoroacetylated (TFA) lysine substrates Boc-Lys(Ac)-AMC or Boc-Lys(Tfa)-AMC was measured as described under Material and Methods. The assay was performed under standard assay conditions at 30^0^C.


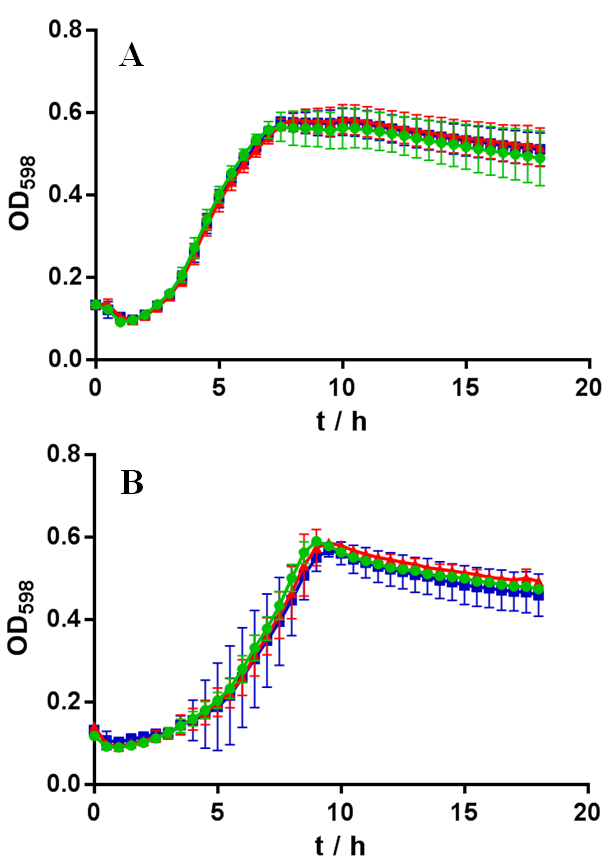


Fig. S2: Growth curves of *P. aeruginosa* wildtypes (PA01: A, PA14: B). All growth curves were performed in the presence of glucose and in the absence of inhibitors (blue squares), in the presence of 50 µM SAHA (red triangle) or in the presence of 50 µM SATFMK (green dots). The data points represent 3 biological replicates with indicated standard deviation.
